# Overview on Sobemoviruses and a Proposal for the Creation of the Family *Sobemoviridae*

**DOI:** 10.3390/v7062761

**Published:** 2015-06-15

**Authors:** Merike Sõmera, Cecilia Sarmiento, Erkki Truve

**Affiliations:** Department of Gene Technology, Tallinn University of Technology, Akadeemia tee 15, 12618 Tallinn, Estonia; E-Mails: cecilia.sarmiento@ttu.ee (C.S.); erkki.truve@ttu.ee (E.T.)

**Keywords:** sobemovirus, taxonomy, pathology, particle structure, genome organization, VPg, polyprotein processing, satellite RNA, virus transmission, RNA silencing suppressor

## Abstract

The genus *Sobemovirus*, unassigned to any family, consists of viruses with single-stranded plus-oriented single-component RNA genomes and small icosahedral particles. Currently, 14 species within the genus have been recognized by the International Committee on Taxonomy of Viruses (ICTV) but several new species are to be recognized in the near future. Sobemovirus genomes are compact with a conserved structure of open reading frames and with short untranslated regions. Several sobemoviruses are important pathogens. Moreover, over the last decade sobemoviruses have become important model systems to study plant virus evolution. In the current review we give an overview of the structure and expression of sobemovirus genomes, processing and functions of individual proteins, particle structure, pathology and phylogenesis of sobemoviruses as well as of satellite RNAs present together with these viruses. Based on a phylogenetic analysis we propose that a new family *Sobemoviridae* should be recognized including the genera *Sobemovirus* and *Polemovirus*. Finally, we outline the future perspectives and needs for the research focusing on sobemoviruses.

## 1. Introduction

The genus *Sobemovirus*, unassigned to any family, was officially included in the plant viral taxonomy twenty years ago. Its name is derived from its type species *Southern bean mosaic virus* (SBMV). In 1969, single-component-RNA beetle-transmitted viruses were proposed to be classified into a southern bean mosaic virus group [1]. In 1977, Hull recommended establishment of this plant virus group on the basis of similarities in capsid properties, molecular weight of genomic RNA and capsid subunit, as well as distribution of particles within the cell [2]. Thereafter, more and more viruses sharing similar properties were included into this genus. The International Committee on Taxonomy of Viruses (ICTV) listed in its last report 14 viruses as definite species [3]. During recent years, some species have been fully sequenced and the new list of sobemoviruses is expected to be longer (Table 1).

**Table 1:** Recognized and putative sobemovirus species.

Species Recognized by ICTV	Abbreviation	Genome Sequence in GenBank (Acc. No)
*Blueberry shoestring virus*	BSSV	-
*Cocksfoot mottle virus*	CfMV	NC_002618
*Imperata yellow mottle virus*	IYMV	NC_011536
*Lucerne transient streak virus*	LTSV	NC_001696
*Rice yellow mottle virus*	RYMV	NC_001575
*Ryegrass mottle virus*	RGMoV	NC_003747
*Sesbania mosaic virus*	SeMV	NC_002568
*Solanum nodiflorum mottle virus*	SNMoV	KC577470
*Southern bean mosaic virus*	SBMV	NC_004060
*Southern cowpea mosaic virus*	SCPMV	NC_001625
*Sowbane mosaic virus*	SoMV	NC_011187
*Subterranean clover mottle virus*	SCMoV	NC_004346
*Turnip rosette virus*	TRoV	NC_004553
*Velvet tobacco mottle virus*	VTMoV	NC_014509
**Related Viruses not Approved by ICTV as Sobemovirus Species**		
*Artemisia virus A*	ArtVA	NC_017914
*Cynosurus mottle virus*	CnMoV	-
*Ginger chlorotic fleck virus*	GCFV	-
*Papaya lethal yellowing virus*	PLYV	NC_018449
*Rottboellia yellow mottle virus*	RoMoV	KC577469
*Snake melon asteroid mosaic virus*	SMAMV	-
*Soybean yellow common mosaic virus*	SYCMV	NC_016033

## 2. Geographical Distribution, Host Range, Transmission

Some sobemoviruses are distributed throughout the world whereas others are limited to only one continent or even to just one endemic region. In some cases, global exchange of infected material and introduction of novel crops in existing or new agricultural areas have expanded virus distribution. Lately, *Sowbane mosaic virus* (SoMV) was imported from Netherlands to Greece [4]. Similarly, the first infections of SBMV were reported in Spain in 2003, most probably due to the introduction of seeds from infected plants [5]. Interestingly, it has been suggested that *Subterranean clover mottle virus* (SCMoV) may have been introduced to Australia following European colonization. The argument for its Mediterranean origin is that SCMoV has a beetle vector there but not in Australia [6]. Similarly, detection of *Cocksfoot mottle virus* (CfMV) in New Zealand is most probably of foreign origin as it has no beetle vector there [7] and its host plant cocksfoot has been introduced [8]. During recent decades, CfMV has colonized new native host species in New Zealand [9].

The natural host range of each virus species is relatively narrow, including monocotyledonous or dicotyledonous plant species. Several sobemoviruses are economically important pathogens. *Papaya lethal yellowing virus* (PLYV) causes serious chlorosis and is responsible for severe disease of papaya in northeast Brazil [10,11]. In Australia, SCMoV decreases clover seed and herbage production [6]. *Rice yellow mottle virus* (RYMV) causes one of the most damaging and rapidly spreading diseases of rice in Africa, producing yield losses that fluctuate between 10% and 100%, depending on plant age prior to infection, susceptibility of rice variety, and environmental factors [12]. The epidemics developed in eastern Uganda a few years ago, with a disease incidence exceeding 70%, were caused by an emergent RYMV strain [13,14]. The extent of the impact of rice cultivation in Africa on RYMV evolution and spread has been elegantly linked [15].

The main transmission source of sobemoviruses is mechanical wounding of host plants. Different species of insects can be vectors for sobemoviruses. The first identified vectors responsible for transmission of sobemoviruses like SBMV, *Turnip rosette virus* (TRoV), RYMV, *Cynosurus mottle virus* (CnMoV), *Solanum nodiflorum* *mottle virus* (SNMoV), CfMV and *Southern cowpea mosaic virus* (SCPMV) were different leaf-feeding beetles [16,17,18,19,20,21,22]. Earlier, insect vectors (a garden flea-hopper *Halticus citri*, a pea leafminer *Liriomyza langei*, a beet leafhopper *Circulifer tenellus* and a green peach aphid *Myzus persicae*), but not beetles, had been described as vectors of SoMV [23]. Remarkably, the mirid bug was found to act as a natural vector for *Velvet tobacco mottle* *virus* (VTMoV), although this virus was also transmitted experimentally by the coccinellid beetle vectors of SNMoV [24]. Later on, it was shown that also SNMoV, SBMV and SoMV can be transmitted by this mirid bug [25], whereas SBMV can also be transmitted by a moth *Diaphaulaca aulica* [26]. In addition, SoMV has been reported to be transmitted by the onion thrips *Thrips tabaci* [27]. A bird cherry-oat aphid *Rhopalosiphum padi* has been identified as the vector of CnMoV in New Zealand, where no beetle vector has been recorded [28]. A blueberry aphid *Illinoia pepperi* is the only known vector of *Blueberry shoestring virus* (BSSV) [29]. Recently, the extensive search for vectors of RYMV found several species of leaf-feeding grasshoppers and sucking bugs in addition to sundry leaf-feeding beetles [30]. It is noteworthy that although the vector transmission in the case of sobemoviruses has been generalized to take place via beetles, there have been a number of other vector species identified so far. In addition, we can state that phylogenetically closely related sobemoviruses (*cf.* Phylogenesis section and figure therein) may have vectors that belong to different orders. It is worth mentioning that some sobemoviruses (SBMV, SCPMV, SoMV, SCMoV and *Snake melon asteroid mosaic virus* (SMAMV)) are seed-transmissible [23,31,32,33,34]. These viruses reside rather in the seed coat than in the embryo [33,35,36].

In experimental conditions, sobemoviruses are easily transmitted by sap-inoculation. Their particles are typically very stable and with a high thermal inactivation point (near 80–90 °C) [37]. The mechanical transmission seems to be the main source of SCMoV that is transmitted by grazing and trampling of virus-infected clover by livestock, by mowing for hay production, and by vehicle wheels [6]. Similarly, the transmission of RYMV can be favored by some cropping practices [38]. Also, RYMV has been shown to move over short distances by mammals [39] and even by birds [40]. It even spreads by wind-mediated leaf contacts and by soil [41,42]. PLYV particles can be found in water and soil [43]. The transmission by soil-borne debris has been reported for SBMV [44] and for *Artemisia virus A* (ArtVA) [45]. Interestingly, the transmission of SBMV was suggested to be abiotic, as the addition of root knot nematodes (*Meloidogyne* sp.), fungi and other soil biota did not increase infection of bait plant in virus-infested soil [46].

As already mentioned, the main way of transmission for sobemoviruses is mechanical wounding of plants. It is highly possible that the described insect-mediated transmission is just another mechanical transmission. This hypothesis does not rule out that some sobemoviruses might display specific mechanisms for transmission by one or another insect species, but probably this is not the main route of how sobemoviruses move from plant to plant.

## 3. Genomic Organization

**Figure 1:**
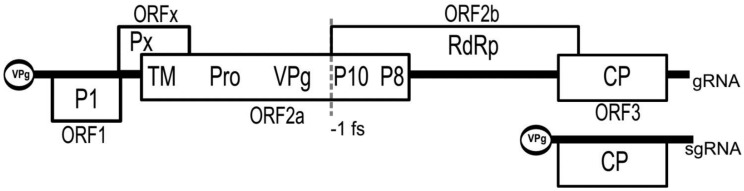
The genome organization of sobemoviruses exemplified by *Sesbania mosaic virus*. P1: first protein; Px: protein “x”; TM: transmembrane domain; Pro: serine protease; VPg: viral genome-linked protein; P10: 10 kDa protein; P8: 8 kDa protein; RdRp: RNA-dependent RNA polymerase; CP: coat protein; gRNA: genomic RNA; sgRNA: subgenomic RNA; –1 fs: -1 ribosomal frameshift. ORF1, ORFx and ORF2a are translated from different frames.

All sequenced sobemoviruses have a polycistronic positive sense single-stranded RNA (ssRNA) genome of approximately 4.0–4.5 kb in size. The 5′ proximal ORFs are expressed from genomic template whereas the most 3′ proximal ORF encoding viral coat protein (CP) is translated from the subgenomic RNA (sgRNA) (Figure 1).

The most 5′ proximal ORF1 encoding protein P1 is situated in weak context for optimal translation initiation [47,48] and this enables pre-bound 40S ribosomes to bypass its start codon and to initiate translation from the subsequent ORF. It has been demonstrated that the deletion of the start codon of SCPMV ORF1 increased translation from downstream ORF2a/2ab, whereas mutations making SCPMV ORF1 translation initiation context more favorable reduced the downstream protein expression [49]. This process is known as “leaky scanning of ribosomes”.

A recent search for “hidden” ORFs in sobemovirus genomes, based on detection of the reduction in synonymous substitutions in gene overlap regions, resulted in the discovery of a region called ORFx [50]. The beginning of ORFx tends to have a non-AUG initiation codon just a few codons before the end of ORF1 (in a good translation initiation context) and it overlaps with the 5′ end of ORF2a 61–88 codons, depending on the species. The functionality of ORFx has been proven by introducing premature termination codons into the ORFx reading frame of an infectious clone of TRoV. Such mutants failed to establish infection [50]. ORFx encodes the so-called protein Px. Both ORFx and ORF1 are the most variable regions in sobemoviral genomes.

For many years, different species of sobemoviruses were divided into two groups according to the organization of their central part of the genome. Later on, it was demonstrated that such variation does not exist and all sobemovirus genomes contain two overlapping ORFs, ORF2a, and ORF2b [51]. Predominantly, sobemoviruses express a polyprotein P2a from the ORF2a. Translation of sobemoviral RNA-dependent RNA polymerase (RdRp) encoded by ORF2b needs a –1 programmed ribosomal frameshifting (PRF) event to switch the polyprotein translation frame after the translation of two thirds of P2a. Studies on CfMV showed that –1 PRF takes place with an efficiency of approximately 10%–20% [52,53]. The –1 PRF signal comprises two elements: a slippery sequence, where the actual reading shift takes place, and a structural element located downstream that greatly stimulates the efficiency of frameshifting. The –1 PRF signal of sobemoviruses consists of the conserved slippery sequence UUUAAAC, followed by a simple stem-loop structure located seven nucleotides downstream of it, with the exception of *Ryegrass mottle virus* (RGMoV) that has 8-nt distance between these elements [52,54]. RNA structure prediction analysis and mapping do not indicate formation of pseudoknots in any of the analyzed sobemovirus sequences in –1 PRF region [54].

The genomic RNA of incoming sobemovirus particles is probably uncoated by the co-translational disassembly mechanism and followed by RNA replication. It has been demonstrated that the particles of SBMV can completely disassemble only after initiation of RNA translation [55,56]. Sobemovirus genome lacks both 5′ cap and 3′ poly(A) tail [57]. Absence of cap and poly(A) indicates that the sobemovirus 5′ and 3′ untranslated regions (UTR-s) must somehow compensate their functions. The 5′UTR bounds covalently a viral genome-linked protein (VPg). The studies on the VPg-s of RYMV, CfMV, SBMV, and RGMoV identified a species-specific linkage between the 5′ phosphate group of the RNA and the hydroxyl group of the amino acid residue (tyrosine, serine or threonine) at the N-termini of VPg-s [58,59]. The RYMV VPg was shown to interact with eukaryotic translation initiation factor eIF(iso)4G [60,61]. This interaction was proposed to serve for ribosome recruitment [61]. In the case of CfMV, it was shown that the 5′UTR itself can operate as a translational enhancer CfMV ε [62]. The CfMV ε is highly successful in the enhancement of reporter genes’ expression in suspension cells of barley—a host of CfMV—compared to other plant virus enhancer sequences like Tobacco mosaic virus (TMV) Ω, crucifer-infecting TMV (CrTMV) IRES, Potato virus X αβ and 5′UTR of Alfalfa mosaic virus RNA4 [62]. All sobemoviral 5′UTRs contain a purine-rich segment also called a polypurine tract [47]. Notably, a polypurine tract was found to be a key element of CrTMV IRES [63].

The 3′ UTRs of sobemovirus genomes show only marginal sequence conservation. A potential tRNA-like structure (TLS) was attributed to the 3′ end of RYMV and CfMV by computer modelling [64,65] but no experimental data is yet available. TLS was not predicted at the 3′ end of SBMV, SCPMV and *Sesbania mosaic virus* (SeMV) [66,67,68]. The non-TLS heteropolymeric 3′ termini have been found customary among plant viruses [69]. Mutational analysis of SeMV 3′ UTR revealed a particular stem-loop structure 28–55 nt upstream from the 3′ end, important for SeMV RdRp template recognition. However, about 20% of the *in vitro* RNA synthesis activity was preserved when the entire 3′ UTR of SeMV was deleted [70]. Studies on SeMV 3′ and 5′ terminal deletion mutants demonstrated that SeMV has the ability to repair 1–5 nt deletions at the genome ends [71]. In addition, studies with CfMV CP deletion mutants indicated that the 3′ UTR might contain sequences or structural elements important for viral RNA transport within the host [72].

Several plant virus VPg-s have been suggested to be involved also in viral replication. Whether the sobemoviral VPg has a role in priming the synthesis of viral RNA is not yet clear. In the case of SeMV VPg, it was shown that it was not required for the *in vitro* negative strand RNA synthesis [70]. Also, no interaction has been identified between SeMV VPg and RdRp *in vitro* [73]. Earlier, a conserved 5′ sequence ACAA(AA) was considered to play a role in viral RNA replication by promoting or enhancing the binding of viral RdRp [74]. Differently from other sobemoviruses, this motif is absent at the genome 5′ ends of CfMV [75] and *Imperata yellow mottle virus* (IYMV) [76]. In the majority of sobemoviral genomes, the ACAA(AA) motif is also present upstream of the translation initiation codon of CP, indicating a possible 5′ terminus of sgRNA [47]. The vicinity of sgRNA transcription start site of SBMV, SCPMV, *Lucerne transient streak virus* (LTSV), RYMV and CfMV is predicted to fold into a hairpin loop. This or its complement in minus-strand RNA is considered to play a role in sgRNA synthesis [47]. In the CfMV sequence, another potentially stable stem-loop complementary to the first one has been predicted to be situated in the middle of the CP encoding region. A hypothetical interaction between these two loops has been proposed to be involved in sgRNA synthesis [77].

The sgRNA has been detected both in sobemovirus particles and in infected tissues [65,75,78,79,80,81,82]. CfMV has also been reported to encapsulate at least five different viral defective interfering RNA molecules (DI RNA) corresponding to 35–40 nt of the 5′ terminus linked to 850–950 nt of the 3′ terminus [83]. Generally, the existence of DI RNA molecules is considered as a proof of the replicase-driven template switching mechanism needed for the creation of recombinant RNA molecules [84].

## 4. Satellite RNAs

In addition to viral RNA, some sobemoviruses encapsidate a viroid-like small (220–390 nt) circular satellite RNA (satRNA) also called “virusoid”. The satRNA is dependent on a helper virus for replication and it can modulate the symptoms caused by the helper virus [85]. The presence of satRNAs has been reported for sobemoviruses like LTSV, SCMoV, VTMoV, SNMoV and RYMV [86,87,88,89,90]. Sobemoviruses like CfMV, TRoV, SBMV and SoMV that normally avoid having their own satRNA, were demonstrated to act as helper viruses for these satRNAs too. The support of replication of satRNA is dependent both on helper virus [91] and on host plant species [92]. Secondary structure models based on thermodynamics predict extensive internal base-pairing of the circular RNAs and suggest a rod-like native structure very similar to that of viroids [93]. Sobemoviral satRNAs replicate through a rolling circle mechanism and self-cleave into monomers by using endogenous hammerhead ribozyme activity [94]. Mutational analysis of LTSV satRNA confirmed that mutations that cause structural perturbations are lethal to satRNA infectivity [95].

Surprisingly, RYMV satRNA that is the smallest satRNA known so far, was reported to express a unique 16 kDa highly basic protein [96]. The mechanism for the 16 kDa protein expression from such “nanogenome” was explained as follows: there is a combined initiation-termination sequence UGAUGA that is accessed via putative IRES. Then, two rounds of translation occur by “shifting” the reading frame at the end of the round, since the satRNA is circular and the number of nucleotides (220) is not a multiple of three. In addition, termination codons can be ignored to obtain even longer read-through proteins [96].

## 5. P1 in Viral Movement and Suppression of RNA Silencing

The expression of P1 of RYMV, SCPMV and CfMV is required for systemic infection [49,81,97]. According to this, P1 was suspected to serve as viral movement protein (MP). The biochemical examination of SeMV P1 showed that it had predominantly α-helical conformation. *In silico* analysis of SeMV P1 suggested the presence of a nucleic acid binding domain and high density of phosphorylation sites in the C-terminal segment. Deletion analysis of SeMV P1, combined with *in vitro* binding assay and with yeast two hybrid assay, showed that the interaction with SeMV P1 and SeMV CP (or particles) was greatly reduced by deleting amino acids at positions 17–49, whereas the C-terminal deletion had a marginal effect on the interaction [98]. Similar results were gained with two other viral proteins—SeMV VPg and P10—which were demonstrated to interact with SeMV P1 via the same region [99]. Accordingly, the N-terminal deletion abolished the interaction between P1 and genomic RNA coupled to VPg. No interaction with genomic RNA was detected when the deletion comprised the C-terminal region, predicted to harbor a nucleic acid binding domain. In parallel, it was observed that SeMV P1 did not recognize the genomic RNA if the VPg had been removed. Hence, it was suggested that the interaction between P1 and VPg might trigger conformational changes in P1, needed to bind directly the genomic RNA via the C-terminal nucleic acid binding domain [99]. The interaction between SeMV P1 and its genomic RNA was found to be highly specific. No interaction was observed when other nucleic acids, including the genomic RNA of an arbitrary virus, were incubated together with SeMV P1 [98]. In a broader sense, the interaction of P1 with the genome-bound VPg and P10 was considered as a cue of an active transport of viral RNA complex facilitated by P1 acting as a MP. The energy needed for that process was suspected to come from the hydrolysis of ATP by SeMV P10, as P1 itself did not show any ATPase activity [99,100]. When expressed in *E. coli*, SeMV P1 appeared to be in large soluble aggregates, characteristic of several MPs [98].

Many MPs act as suppressors of RNA silencing [101] and this is also the case for P1 of sobemoviruses. P1 of RYMV, CfMV and SBMV were characterized as suppressors, although this activity was shown in its host plant only for RYMV P1 [102,103,104,105]. Noteworthy, it is not yet clear if P1 RNA silencing suppressor activity can be uncoupled from the movement function.

Using *Agrobacterium* transient assays to evaluate the suppression of RNA silencing in *Nicotiana benthamiana*, RYMV P1, CfMV P1 and SBMV P1 showed a clear suppressor activity at the systemic level and a weak suppression at the local level [103,105]. However, at least for RYMV P1 the suppression activity cannot be described in a general way, as the P1s from analyzed isolates act differently. Indeed, the previously mentioned result is valid for P1of RYMV-Ni isolate, but P1of RYMV-Tz3 and of RYMV-Mg1 isolates do not suppress systemic silencing, on the contrary, they enhance it. This means, that these P1 proteins modulate the RNA silencing host response enhancing and suppressing it at different levels, presumably seeking a successful infection by maintaining equilibrium between efficient virus multiplication and preservation of the host integrity [106]. This important dual activity remains to be demonstrated in rice.

When expressed transgenically in *N. benthamiana*, RYMV P1 affected the normal plant phenotype whereas CfMV P1 did not. Concurrently, both suppressors enhanced the spread but not the accumulation of CrTMV [107]. Transgenic rice expressing P1, either from RYMV-Tz3 or RYMV-Mg1, displayed inflorescence developmental defects comparable to those previously described in a rice mutant line with a deletion in the Dicer-like 4 gene (OsDCL4; [106,108]). It was further demonstrated, that P1 transgene specifically affected the endogenous small interfering RNA (siRNA) pathway dependent on DCL4 [106]. At the molecular level, it is not known how the different small RNA pathways are influenced during suppression by P1. It was shown that CfMV P1 does not bind siRNAs [103] although it is known to bind ssRNA in a sequence-independent manner [109].

**Figure 2:**
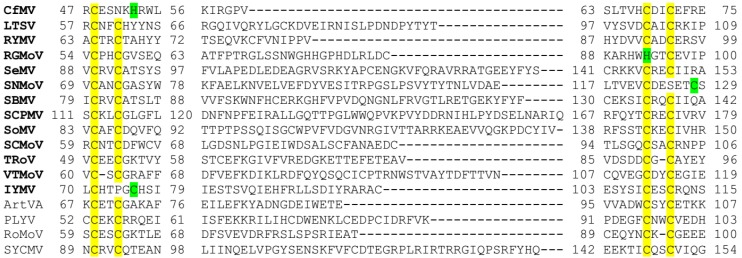
CLUSTAL O (1.2.1) multiple sequence alignment (manually adjusted). Sobemoviruses recognized by ICTV (bold); conserved cysteine residues of putative zinc-finger motif (yellow); histidine or cysteine residues close to the conserved position (green). The central part is not aligned because no homology is detected.

The investigations on the diversity of P1 silencing suppressor activity of a set of RYMV isolates showed that the capacity to suppress silencing is not linked to pathogenicity or phylogeny [104]. Variations in silencing suppression strength were correlated to specific amino acid residues. Mutagenesis in P1 sequence demonstrated the importance of the first and last cysteine residues of the putative zinc-finger (Znf) motif [C64-X_2_-C67-X_24_-C92-X_2_-C95] for the suppression of RNA silencing and cell-to-cell movement ability of P1 [104]. Notably, Znf motifs were previously reported to be related to silencing suppression activity of other viral proteins [110,111]. Biochemical studies on P1 of RYMV-Tz3, the strongest P1 suppressor of local silencing, revealed a second possible zinc-binding domain including residues [H109-X_34_-C140-X_4_-H145-X_3_-C149], and most importantly, it was shown *in vitro* that P1 reversibly binds two zinc atoms in a redox-dependent manner. In addition, a conformational change in P1 was observed upon exposure to oxidative environment (H_2_O_2_), suggesting a biological role during viral infection connected to P1 conformations and its dual function in silencing. Interestingly, oxidized P1 accumulates as monomers and as oligomers [112]. It is worth mentioning, that the four essential cysteine residues involved in the formation of the first Znf motif are not only conserved among all RYMV P1 isolates, but also among sobemoviruses in general. Figure 2 shows the alignment of the just mentioned region of all sobemoviruses. Few viruses harbor only three cysteine residues at the conserved positions, but another cysteine or histidine residue is close-by, suggesting that the Znf motif is indeed conserved. Other isolates of the different viruses were checked and no significant differences were observed. It should be noted that the homology among P1 proteins is hardly detectable since the percentages of identity or similarity are low (e.g., 8% identity between SeMV P1 and CfMV P1; [98]).

Lately, 54 different RYMV isolates were analyzed to find out sites in the P1 sequence that are under positive selection. A big number (18) of sites was found and some of those were validated with mutagenesis for their role in suppression of RNA silencing. The results showed that P1 evolved rapidly, reflecting the importance of the suppressor for the adaptation of the virus [113].

Interestingly, the N-terminal half of RYMV P1 was found to possess an autocatalytic activity most probably designated to keep the original C-terminus of P1 [114]. Whether this is characteristic of other sobemoviral P1s, remains to be elucidated.

Remarkably, CfMV has in addition to P1 also CP acting as suppressor of RNA silencing [72]. In fact, there are a number of viruses coding more than one suppressor [115,116]. Both suppressors, P1 and CP, interfere with the RNA silencing mechanism independently and a strong synergistic effect has not been observed [72].

## 6. Proteolytic Processing of Polyprotein

In addition to P1, Px and CP, viruses need several other proteins with different functions. In sobemoviruses these proteins are translated as a polyprotein. Two versions of polyprotein having a different C-terminus are translated from the central ORFs 2a and 2ab. The mutual N-terminal part of the sobemoviral polyproteins P2a and P2ab consists of the N-terminal membrane anchor, the serine protease (Pro) and VPg domains. The C-terminal part of P2a is not conserved. It was demonstrated that SeMV P2a C-terminus contains an RNA-binding domain (P10) and a novel ATPase domain (P8) [100]. The C-terminal part of the polyprotein P2ab consists of the motifs characteristic for an RdRp. The position of VPg between the viral protease and RdRp is unique among the phylogenetically related sobemo-, polero-, enamo- and barnaviruses [117,118,119,120]. Sobemovirus polyprotein undergoes proteolytic processing carried out by its own serine protease [121]. The protease catalyzes hydrolysis of specific peptide bonds located between two specific amino acid residues. Identification of sobemoviral VPg-s attached to the viral genomes indicated that the polyprotein is processed at E/T residues for SBMV, at E/N and E/T residues for CfMV, at E/S and E/T residues for RYMV and at E/S residues in the case of RGMoV. The VPg C-terminal cleavage site was found to locate upstream of the –1 PRF elements [58,59].

Sequence alignments showed similarity between sobemoviral proteases and the ones from polero-, enamo-, and barnaviruses [122]. The proposed consensus sequence is H(X_32–35_)[D/E](X_61–62_)TXXGXSG, where H, D/E and S constitute a catalytic triad and X denotes any amino acid [123,124]. Mutational analysis of the active site residues (H181, D216, and S284) in SeMV protease verified their crucial role in protease activity. SeMV is the only sobemovirus for which all polyprotein processing sites have been characterized [100,121]. Namely, four cleavage sites have been found and validated. Analysis of mutants confirmed the cleavage at E/T residues at both sides of VPg, as well as the cleavage at E/S residues between the N-terminal domain and Pro, but also within the C-terminal domain of P2a.

The structure of the SeMV crystallized Pro domain has been determined at a resolution of 2.4 Å. Remarkably, a comparison of the 3D-structure of SeMV protease domain with all the available entries in the Protein Data Bank indicated that it is closer to the non-viral proteases than to the viral ones [125]. The structure of SeMV protease exhibits the characteristic features of trypsin fold with a well-formed active site and a substrate-binding cleft. It consists of two β-barrels connected by a long inter-domain loop. Both the active site and the substrate-binding cleft are located between the two barrels and are fairly exposed to a solvent. Mutation analysis of glutamate-binding site (S1-binding pocket) residues H298, T279 and N308 of SeMV Pro demonstrated that these are indeed crucial for protease activity. Also, several downstream residues were shown to be important for the protease activity [125]. The substrate specificity of SeMV protease was predicted as N,Q-E/T,S-X (where X is an aliphatic residue) [100]. However, the multiple sequence alignments revealed that there is no common substrate specificity for all sobemoviruses, except that the S1 binding pocket seems highly specific for glutamate or glutamine [126].

The cleavage between the N-terminal domain and the protease is crucial for the efficient processing of SeMV polyprotein (both P2a and P2ab), in particular for the cleavage within the C-terminal domain of P2a. These two cleavage sites are only accessible by *in cis* auto-proteolysis [71,100]. Indeed, the relative positions of the N-terminal cleavage site and the active site in the crystallized protease domain suggest that such intramolecular proteolysis is possible [125]. The N-termini of all sobemoviral polyproteins have been proposed to contain transmembrane helixes [126]. Therefore, the N-termini putative function is to anchor the polyprotein into cellular membranes to facilitate proteolytic processing and probably also viral minus strand synthesis [100]. Interestingly, the multiple sequence alignment of the P2a proteins of phylogenetically related sobemo-, polero-, polemo-, enamo- and barnaviruses suggests the presence of structural constraints that strictly determine the distance between the N-terminal cleavage site and the protease active center [126].

Significant accumulation of the precursor protein Pro-VPg was observed in membrane fractions of SeMV infectious cDNA infiltrated leaf samples [71]. Pro-VPg, but not protease alone, is crucial for both the *cis* and *trans* catalytic activities of SeMV protease. Most probably, a release of free Pro domain from Pro-VPg during the proteolytic self-processing changes the conformation of the protease in a way that it cannot cleave the substrate any more. The activity of Pro-VPg has been shown to confer aromatic stacking interactions between W43 of the VPg and W271 and H275 of the Pro domain [127,128].

The proteolytic processing of viral polyproteins is a finely tuned action, as different domains carry activities needed for different steps during the viral life cycle. The studies on SeMV revealed that the polyproteins with mutual N-termini (P2a and P2ab) undergo proteolytic processing with different dynamics. Whereas P2a is the source of active Pro-VPg, Pro domain appears to be released when P2ab is expressed. The proteolytic processing of P2ab leads to the accumulation of the precursor protein VPg-RdRp [100]. The primary studies on VPg-RdRp suggest that it has an inhibitory effect on the *in vitro* polymerase activity [100].

## 7. The Proteins VPg, P8, and P10

The only conserved sequence element observed among the VPg-s of sobemo-, polero-, enamo- and barnaviruses is a WAD/WGD/WNK motif followed by a D/E-rich region [59,129].

SeMV VPg tends to be an intrinsically disordered protein [127]. These kinds of proteins are believed to adopt a rigid conformation stabilized *in vivo* upon interaction with natural substrates [130]. This could be the case also for the other sobemoviral VPg-s. Indeed, according to the predictions of sobemo-, poty- and caliciviral VPg-s, it was proposed that intrinsic disorder is a common feature of the VPg-s that confers on them the ability to bind many different partners and to fulfil different functions during the viral life cycle [131]. The VPg-s of RYMV, CfMV, RGMoV and SBMV contain several phosphorylated residues, most probably related to the regulation of folding and unfolding of disordered VPg proteins during and for interaction determination [58,59]. SeMV VPg has been shown to interact with its own P1 but not with its RdRp; other interaction partners have not yet been tested [73,99].

RYMV VPg was identified as a virulence factor [60]. It interacts directly with the central domain of rice eIF(iso)4G1 [61,131]. The mutations in eIF(iso)4G1 corrupt the interaction and correspond to the *Rymv1* resistance alleles (*cf.* Pathology and Resistance section). Several populations of RYMV strains (so called virulent strains) were observed to overcome the *Rymv1* resistance, most often by mutating VPg in the codons 48 and 49. In avirulent isolates, position 48 was occupied by a conserved arginine, whereas this site is polymorphic (having glycine, isoleucine or glutamic acid) in virulent isolates [60,132]. Also, threonine at position 49 has been characterized as the genetic constraint blocking the emergence of resistance breaking mutations [133]. Resistance breaking phenotypes have also been associated with codons in other positions [61,131,132,133,134].

Surprisingly, few *Rymv1* resistance breaking RYMV variants seem to be related to mutations in protein P8 homolog encoded by the C-terminal part of ORF2a [135]. Similarly to the VPg-s, the P8 proteins of RYMV and SeMV display a disordered arrangement in prediction analyses [100,135]. Therefore, it was suggested that the RYMV P8 can be involved in the interaction between RYMV VPg and the eIF(iso)4G1 of rice [135]. SeMV P8 contains an RNA binding region and is able to bind RNA and DNA *in vitro* [100]. The RNA-binding motif was predicted to be in the C-terminus of CfMV P2a [54]. In addition, SeMV P8 can be phosphorylated. It is also responsible for the Mg^2+^-dependent ATPase activity of P10 in the precursor protein P10–P8 [100].

SeMV P10 was shown to interact strongly with P1 and RdRp; other possible partners have not yet been tested [73,99]. While the interaction of P10 with P1 is believed to be involved in the formation of a viral movement complex, the interaction with RdRp is expected to be related to virus replication. *In silico* analysis of SeMV P10 suggests that it possesses random coils at N- and C-termini and an α-helix in the middle. The random coils, like disordered proteins, undergo conformational changes and acquire a secondary structure upon protein-protein interaction [73].

## 8. RNA-Dependent RNA Polymerase

The RdRp of sobemoviruses was identified via a highly conserved GDD motif (SGSYCTSSTNX_19–35_GDD) that is characteristic of RdRp-s of positive-strand ssRNA viruses [122]. According to sequence similarities, RdRp-s of sobemo-, polero-, enamo- and barnaviruses are classified as a “sobemo-lineage” in the supergroup I of plus-sense plant RNA viruses. The RdRp-s of this group, as well as some of supergroup II (luteoviruses and viruses from the family *Tombusviridae*), apparently lack the conserved NTP-binding elements characteristic of viral helicases [136,137]. Studies on the recombinant RdRp of SeMV confirmed that the GDD motif is indeed essential for the activity of SeMV RdRp. It has been demonstrated that SeMV RdRp can synthesize RNA in a primer independent manner and that the synthesized end product is double-stranded RNA not covalently linked to the template [70]. The co-expression of SeMV RdRp and P10 results in significantly higher polymerase activity than in the case of recombinant RdRp alone. Not surprisingly, SeMV RdRp interacts with P10 over the disordered C-terminal region of RdRp. The disordered state of the C-termini of RdRp-s is conserved across the genus *Sobemovirus*. In addition to the interaction between SeMV RdRp and P10, a moderate interaction was revealed between SeMV RdRp and Pro. The roles of these interactions remain to be elucidated. The *in vitro* tests did not show RdRp interaction with VPg, CP, or P8 [73].

## 9. Virion Topology

**Figure 3:**
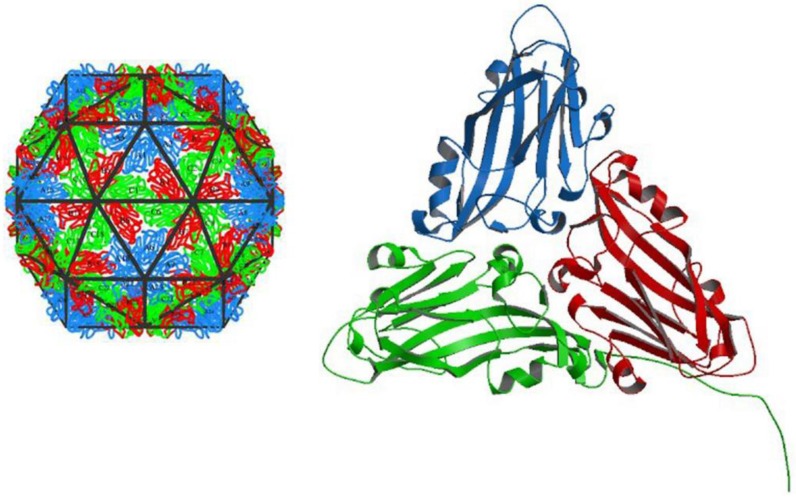
Cocksfoot mottle virus capsid. Sobemovirus capsid is assembled according to T=3 icosahedral lattice symmetry. The CP monomers are chemically identical but exist in three slightly different conformations, denoted as A (blue), B (red) and C (green) subunits. The A, B and C subunits build one icosahedral subunit (on the left). Altogether, there are 60 icosahedral subunits per sobemoviral T=3 particle (on the right). The A subunits interact at the icosahedral fivefold axes to form 12 pentamers while the pairs of B and C subunits meet at icosahedral threefold axes to form 20 hexamers. The pentamers and hexamers differ significantly in shape—hexamers are generally planar and pentamers are substantially bent. The combination of hexamers and pentamers gives the particle its characteristic shape. Pictures taken from http://viperdb.scripps.edu [146].

The virions of sobemoviruses have an icosahedral capsid with an approximate diameter of 30 nm (Figure 3). The capsid consists of 180 molecules of a single 26–34 kDa CP translated from sgRNA [57]. The 3D structures of SCPMV [138], SeMV [139], RYMV [140], CfMV [141], and RGMoV [142] virions were determined utilizing X-ray crystallography. Despite the fact that primary sequences of sobemoviral CPs are quite different (the sequence similarities between the CPs of previously mentioned sobemoviruses are 12%–30% except the 63% similarity between SeMV and SCPMV, earlier considered as isolates of one virus species), their 3D structures are nearly identical. Actually, it is a general observation that 3D structures of structural proteins are better conserved than their amino acid sequences [143]. The root mean square (rms) deviations between superimposed coordinates of Cα atoms of the respective sobemoviral CP residues are in general 1.4–1.5 Å [142]. Interestingly, RGMoV seems to be slightly different from other sobemoviruses—rms deviation between the superimposed coordinates of Cα atoms is respectively 1.8–1.9 Å. Its virion is smaller than the virions of other sobemoviruses and it is slightly more similar to the virion of *Tobacco necrosis virus* (TNV-A) from the family *Tombusviridae*, genus *Alphanecrovirus* [142]. Indeed, according to the sequence similarities, CPs of sobemoviruses are most closely related to those of necroviruses [144]. The sequence similarity between TNV-A CP and sobemoviral CPs is 15%–27% and rms deviation between the superimposed coordinates of Cα atoms of the respective residues of sobemoviral CPs is 1.4–1.5 Å [142]. The 3D homology modelling of the CP of the recently sequenced *Rottboellia yellow mottle virus* (RoMoV) indicates the overall fold characteristic of RGMoV CP [145]. The identification of ArtVA revealed another sobemovirus closely related to RGMoV [45].

Studies on TRoV, SCPMV, SeMV, and RYMV particles demonstrate that the stability of the virions depends greatly on pH and the availability of divalent cations, Ca^2+^ and Mg^2+^ [37,147,148,149]. Upon alkaline pH or removal of the cations, the virus particles swell and become less stable. The radius of RYMV and SCPMV particles increases about 7% during the swelling process [37,150]. It has been proposed that removing the Ca^2+^ ions results in electrostatic repulsions that trigger the swelling [151]. Calcium binding sites are located between the subunits AB, BC, and CA. Sobemoviral particles were shown to bind three Ca^2+^ ions per icosahedral subunit ABC, *i.e.*, 180 Ca^2+^ ions per particle [152]. A structure-based alignment of the sobemoviral CP sequences identified eight invariant amino acid residues. Three of these involved binding of the Ca^2+^ ions, others were suggested to be important for the backbone conformation [142]. Mutational analysis of SeMV CP calcium binding sites demonstrates that cation-mediated interactions are mainly needed for particle stability but not for assembly [149]. The binding of calcium contributes to rigid packing of protein subunits into the viral particle [149,153]. Depending on the pH and the presence or absence of calcium, RYMV particles have been demonstrated to exist in three forms. The unstable swollen form is present in the cytoplasm from where it is proposed to move into the vacuoles for compaction. It has been proposed that a swollen particle might be an intermediate state before disassembly and after assembly of virions *in vivo* [149,151]. In infected rice plants, transitional and swollen forms were abundantly found during early infection, whereas compact forms increased during later stages of infection [154].

In addition to external factors such as pH and divalent metal ions, sobemovirus particles are stabilized by protein-protein interactions between subunits and by CP-RNA interactions [37]. A single mutation W170K in SeMV CP has been found to result in stable CP dimers not assembling into particles [155]. The assembly of SBMV and SeMV virions was proposed to be initiated by a pentamer of dimeric units or 10-mer at the icosahedral 5-folds [156,157,158]. Further assembly was proposed to proceed in the presence of RNA [159]. It is presumed that packing of viral nucleic acid requires recognition of a specific region within the virus genome by viral CP. In the case of SCPMV, a putative stem-loop sequence (mapped to the position corresponding to nt 1410–1438) within a conserved region encoding serine protease has been reported to bind CP [160]. However, it has not been demonstrated to nucleate SCPMV assembly.

The monomers of sobemovirus CP possess an eight β-strand anti-parallel twisted sheet conformation known as a jellyroll β-sandwich or β-barrel topology, common in most non-enveloped icosahedral viruses [161]. In general, sobemoviral CP has two domains: N-terminal R (random) domain that is found to be completely disordered in subunits A and B but partially ordered in subunit C; and C-terminal S (shell) domain, which is the core building block of the virion [138,152,156]. It has been proposed that the disordered beginning of R domain interacts with RNA in the interior of the virus particle [138,162]. The partially ordered N-terminal arm of C subunit is inserted between the interacting sides of the subunits, making the contacts between subunits flat (*i.e.*, tensed state). The contacts between the subunits that lack the inserted arms are bent (*i.e.*, relaxed state). In that manner, the N-terminal arm acts as a molecular switch regulating the curvature of viral capsid and the assembly of T=3 particles. The removal of the R domain from CP results in the formation of T=1 particles (composed of 60 identical monomers), with only bent contacts between subunits [157,159]).

The first half of R domain (*ca.* 30–35 amino acid residues) contains a conserved arginine-rich motif (ARM), whereas the following half is responsible for the formation of structure called β-annulus, which realizes only in case of partially ordered N-terminal arms of C-subunits. The analysis of SeMV CP has shown that deletion of the first part involving ARM results in the formation of T=1 and pseudo T=2 particles [153,157]. On the contrary, deletion of the amino acid residues that constitute the β-annulus do not affect T=3 capsid assembly or stability [158]. Moreover, the assembly of such kinds of SeMV particles takes place without the formation of the β-annulus that may be therefore formed only as a consequence of the particle assembly [163]. SCPMV, SeMV, and RGMoV have been found to be more sensitive to pH elevation than RYMV and CfMV. The difference is associated with the slight differences observed in the arrangement of β-annuli of the N-terminal arms of C-subunits [140,141,142,151]. Amino acid residues forming the inter-subunit contacts are not conserved among sobemoviral CPs [141,151].

R domain is rich in arginine, lysine, proline, and glutamine, which have been considered to be responsible for coat protein contacts with RNA [152,156]. It has been shown that the ARM of CP are determinant for a nonspecific *in vitro* RNA binding activity [164]. The overall charge of the ARM, but not the arginine residues at specific positions, is responsible for RNA binding in SCPMV and SeMV [158,164]. If only the arginine residues of ARM are replaced with glutamic acid residues, formation of empty T=3 particles with reduced stability takes place. Hence, ARM is prerequisite for RNA interaction and encapsulation [158]. The presence of RNA has been shown to enhance the overall stability of the virion [158]. Studies on sobemoviral CP-RNA interactions do not demonstrate the requirement for specific interactions. Similarly to viral RNA, RNA of bacterial origin (23S rRNA or its degraded variants) is packed into SeMV particles when expressed in *E. coli* [157]. CP of CfMV shows rather a general affinity to bind any kind of ssRNA as well [109]. The ARM contains a bipartite nuclear targeting signal (NLS) [64,165]. Abolishment of the NLS does not affect CfMV infectivity [72]. In addition to RNA-binding properties, the highly basic region of the N-terminus of SCPMV CP (involving ARM) withholds a potential to form α-helix and it has been shown to interact with artificial membranes *in vitro* [166]. The actual biological relevance of this membrane interaction is not known.

Interestingly, it has been postulated that nonspecific electrostatic interactions might control both the genome length and conformation of all ssRNA and ssDNA viruses with highly basic peptide arms of CPs. As a result of mathematical modelling, a genome length was found to be linear in the net charge of CP peptide arms but not with the geometry and volume of the virion [167]. Albeit the calculated genome packing density is similar within the sobemovirus group, it varies significantly among different families of small ssRNA viruses [142].

## 10. Virion Assembly and Disassembly

A large number of studies have allowed the proposal of a model for the sobemovirus virion assembly. First, the CP subunits with disordered amino termini assemble into a pentamer of AB dimers. Interaction with the amino terminal ARM with RNA leads to the formation of CC dimers and ordered β-annulus. Subsequent addition of CP dimers leads to the formation of swollen T=3 particles. The particles become compact after the addition of calcium ions at the subunit interfaces [168].

Virion disassembly starts by swelling after removal of calcium ions. RYMV and SBMV particles show considerable structural changes particularly at the pentamer centers [151,169]. Analysis of the 3D structure data on the SBMV virion shows that the centers of pentamers form long channels bearing some homology with the nicotinic acetylcholine receptor channel [170]. The modelling of the ion-protein interaction energies suggests that this channel is attractive for cations. It was speculated that the channels providing cations ensure electrical neutrality of the RNA via stabilization of the inner viral media, or *vice versa*, they are related to destabilization of the capsid [170]. Calculation of the distribution of elastic constants and of yielding forces within the SBMV capsid observed weakening along the fivefold symmetry axes, leading to the suggestion that pentamers are possible exit ports for RNA release. It was assumed that genome release is preceded by an opening of capsomers instead of a complete capsid bursting [169]. Swollen SBMV particles seem to release their genomes by interaction with ribosomes. Further removal of coat protein subunits occurs as ribosome translocation on viral RNA proceeds [55,56].

Systemic movement of SCPMV [171] and RYMV [172] is dependent on correct particle formation. Moreover, the RYMV CP transgenic rice enhances infection of RYMV [173]. TRoV CP has an ability to complement the systemic movement of a taxonomically distinct virus, red clover necrotic mosaic dianthovirus [174]. The protein interaction studies on SeMV, the close relative of SCPMV, show that both CP and the native virions of SeMV interact with P1 [98], implicated in virus movement, as explained before. Oppositely, CP-deficient CfMV spreads within a host plant successfully although it is not transmittable mechanically by sap-inoculation [72]. Therefore, it was concluded that particle formation is needed only for efficient transmission of CfMV differently from SCPMV and RYMV. In addition, the fact that CP of CfMV is not needed for cell-to-cell or for systemic movement means that this sobemovirus moves as RNP complex and not as virion. Thus, individual sobemoviruses use different trafficking strategies [72].

The virus particle formation is also important for vector transmission due to the high RNase activities in regurgitant of leaf-feeding beetles who can transmit sobemoviruses in a semipersistent manner [175]. SBMV and SCPMV can be retained for several days in the haemolymph of bean leaf beetle, spotted cucumber beetle, and Mexican bean beetle [176]. Infective VTMoV particles have been found also in the feces of mirid bug *Cyrtopeltis nicotianae* six days after acquisition [177].

## 11. Localization of Virions in Cells and Tissues

Sobemoviral particles have been detected in the cytoplasm, nuclei and in vacuoles. Studies with RYMV suggest that vacuoles become the storage compartments for virions in the course of infection. It is proposed that swollen and less compact virions exist in the cytoplasm, whereas vacuoles with acidic pH and higher Ca^2+^ concentration contain compact virions [151,172]. Late in infection, particle accumulation results in large crystalline aggregates and inclusions in the cytoplasm and vacuoles [178]. The formation of inclusions can be dependent on the tissue type invaded. For example, crystalline arrays of RYMV have been observed in vascular tissues but normally not in mesophyll cells [154,172].

It has been suggested that several sobemoviruses can move into the nucleus as virions thanks to nuclear targeting signal of CP [64,72]. Sobemoviral particles are usually not detected in mitochondria and chloroplasts. However, the chloroplasts of cells infected with SBMV [179] or RYMV [172] have been reported to form sometimes finger-like extrusions. When RYMV particles are not stored in vacuoles but appear in large quantities in the cytoplasm, then degenerative changes occur within chloroplasts in mesophyll cells [154]. Other cellular changes include proliferation of tonoplast membranes bulging into the vacuole in SoMV-infected [180] or CfMV-infected [181] host cells.

Studies on tissue distribution of sobemoviral particles have revealed them in leaf mesophyll, epidermis and palisade cells, in guard cells of stomata, in vascular tissues (both in xylem and phloem) and in bundle sheath cells surrounding them, and even in meristem cells. Distribution patterns of individual sobemoviruses differ to some extent [182].

In rice, RYMV particles have predominantly been detected in xylem parenchyma and vessels, in bundle sheath cells and leaf mesophyll. In the late stage of infection, RYMV particles have been observed only occasionally in the phloem. It has been proposed that the virus is transported between xylem cells by binding calcium from pit membranes into the composition of virion [151,154,172]. A high stability of virions is required for translocation via xylem because of the action of proteases during programmed cell death of tracheary elements [183,184]. Also SoMV, BSSV, and SMAMV have been observed rather in xylem than in phloem [34,180,185,186]. However, CfMV, SBMV, and SCPMV particles have been found predominantly in phloem [179,187,188,189]. In the early stage of infection, CfMV particles were detected in phloem parenchyma and bundle sheath cells and later in mesophyll cells surrounding vascular bundles and only seldom in xylem parenchyma [189].

## 12. Pathology and Resistance

The external outcome of sobemoviral infections varies from mild to severe chlorosis and mottling; also stunting, necrotic lesions, vein clearing and/or sterility have been documented [182]. However, some infections have been reported to be symptomless [190,191,192].

Virus infection causes major rearrangements on host physiology. RYMV induces enlargement of nucleolus and disorganization of the middle lamellae of the cell walls of parenchyma and mature xylem cells [172]. Interestingly, RGMoV was reported to induce apoptotic cell death in oat leaves [193]. According to the data gained from purification of RYMV-host protein complexes *in vivo* and *in vitro*, the virus infection interferes with the host metabolism, defense, and protein synthesis [194]. For instance, the expression levels of several defense- and stress-related proteins like superoxide dismutase and different heat shock proteins increase several times [195].

In general, production of reactive oxygen species and antioxidant metabolism are figured to be involved in symptom development and pathogenesis in plant-virus interactions. The measurements of reactive oxygen species and antioxidant enzymes of cocksfoot plants susceptible to CfMV and plants with acquired immunity to CfMV, show completely different patterns in up- and down-regulation after inoculation of CfMV. For example, in susceptible plants, H_2_O_2_ levels declined immediately after inoculation with CfMV and then gradually increased. Increase in H_2_O_2_ levels induced elevated lipid peroxidation and symptoms development. Conversely, recovered plants resistant to a new infection showed only a brief increase in H_2_O_2_ levels immediately after inoculation, with no significant increase in lipid peroxidation [196].

Natural resistance to sobemoviruses has been detected for CfMV in cocksfoot [197,198], for CnMoV in *Cynosurus cristatus* [197], for RYMV in *Oryza sativa* [199,200,201] and in *O. glaberrima* [18,199,201,202], for SBMV in beans [203], for SCPMV in cowpea [204] and for SCMoV in subterranean clover [205,206].

The molecular mechanisms conferring resistance have been described only for RYMV in *Oryza* species. Namely, a monogenic recessive resistance trait *Rymv1* [201] was mapped on chromosome 4 [207]. *Rymv1* has been identified to encode eIF(iso)4G [208]. *Rymv1-1* allelic variant is characteristic of susceptible varieties, whereas four other allelic variants are related to different levels of resistance against RYMV. *Rymv1-2* was found in *O. sativa*, while *Rymv1-3*, *Rymv1-4* and *Rymv1-5* are three distinct resistance alleles in *O. glaberrima*, an indigenous African rice species [208,209]. All these resistance-conferring allelic variants are suggested to be a result of convergent evolution [12,208]. The difference between *Rymv1-1* and *Rymv1-2* lies in one amino acid substitution (E309K) in the central region of the eIF(iso)4G gene [208,210]. *Rymv1-2* resistance does not confer a strict immunity, but it allows limited replication and systemic movement of the wild type RYMV genotype [60]. Breakdown of the resistance conferred by *Rymv1-2* has been reported for some RYMV isolates [38,132] due to non-synonymous mutations in RYMV VPg [211]. However, substitutions in RYMV VPg that were observed to enable overcoming of the *Rymv1-2* resistance did not operate in *Rymv1-4* plants [208,211]. Similarly, only a small subset of RYMV VPg mutants breaking the resistance of *Rymv1-3*, were able to overcome the *Rymv1-2* resistance [12]. Nonetheless, the 3D topology and the biochemical properties of virulence mutations both suggest a direct interaction between RYMV VPg and rice eIF(iso)4G encoded by *Rymv1* [211]. Besides *Rymv1* that expresses high resistance against RYMV infection, but in limited number of cultivars, there are several other quantitative trait loci (QTLs) associated with partial resistance against RYMV mapped on rice chromosomes 1, 2, 7 and 12 [200,212,213]. Expression of QTL_12_ has been reported to confer partial resistance via delayed movement of RYMV into mestome (bundle sheath cells; [214]). Evaluation of genes from *eIF4E* and *eIF4G* multigenic families as potential candidates for partial resistance QTLs to RYMV in rice identified three members of the *eIF4G* as good candidates, while members of the family *eIF4E* seemed not to be involved in conferring resistance, unlike as described in several other studies on plant-virus interactions [215]. Recently, QTL_1_ was mapped outside the *eIF4E* gene as *Rymv2* [209]. Further analysis showed its association with the rice homolog of *CPR5* (*constitutive expresser of pathogenesis related genes-5*), a regulator of active defense mechanism [216]. The sequencing of the candidate region revealed one nucleotide deletion leading to a truncated and probably non-functional protein.

## 13. Phylogenesis

**Figure 4:**
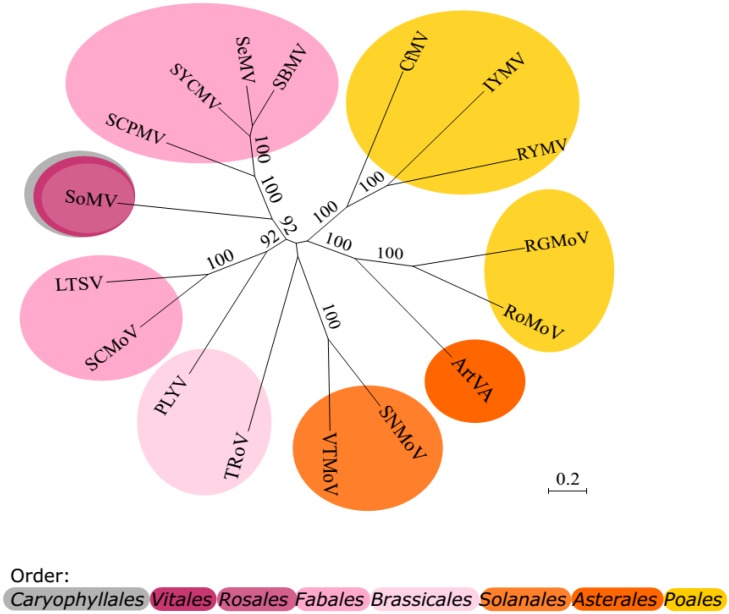
Unrooted maximum-likelihood tree of the complete sobemovirus genome sequences. The tree was generated with the Seaview 4.4.2 program using the GTR nucleotide substitution model [219]. Numbers at the branches indicate bootstrap values (only the scores exceeding 70% are shown). The support of the nodes was assessed after 1000 bootstrap replicates. The scale bar represents the number of nucleotide substitutions per site. Affiliation of the host species to the higher taxonomy units: core eudicots (grey), rosids (pink), asterids (orange), and commelinids (yellow).

It is not clear whether the ancestor of sobemoviruses originates from monocotyledonous or dicotyledonous plant species. The majority of sequenced sobemoviruses infect dicotyledonous hosts. As said before, host ranges are narrow (except for SoMV) and specific for the different sobemoviruses. The genetic relationships are stronger between species infecting plants from the same families. Clades supported by the bootstrapping scores are presented in Figure 4. However, when the individual proteins are compared, the clustering of PLYV and SoMV is not always clear, similarly to TRoV (not shown). Interestingly, the phylogenetic data suggest that commelinid (*Poales*)-infecting sobemoviruses have emerged at least twice during diversification of sobemovirus species [145,217]. The clustering of RoMoV/RGMoV together with ArtVA indicates a possible host-shift from asterid plant species to graminaceous one [45,145]. The same might apply for the groups of CfMV/RYMV/IYMV and VTMoV/SNMoV, according to the analysis of the VTMoV sequence [218].

The large-scale sequencing of RYMV isolates has enabled to date the virus diversification time in *Sobemovirus* genus. The divergence time of RYMV has been found to be *ca.* 200 years, which spans the period of extension of rice cultivation in Africa [217]. It has been calculated that the divergence time among sobemoviruses was approximately 500–3000 years. The divergence with related viruses was calculated using RdRp sequences and it was considered to be around 4000 years for sobemoviruses and MBV, 5000 years for sobemo-, barna-, and poleroviruses, and 9000 years for sobemo-, barna-, polero-, and luteoviruses [217]. This timeline spans the domestication and spread of cultivated plants, raising the hypothesis that the emergence of these plant viruses is linked to the development of agriculture [217]. The calculations made by Pagan and Holmes [220] set the origin of *Luteoviridae* members within the last 4000 years. According to this study, the estimated split of the *Luteovirus* and *Polerovirus* genera took place no earlier than 1500 years ago and all individual luteovirus species appeared within the last 500 years. Although the time scale speculation for the evolutionary changes is shorter than the one proposed before, it is still linked to the development of agriculture.

A mean mutation rate of RYMV genome has been calculated to be 5.2 × 10^−4^ nt/sites per year. However, a similar result (6.83 × 10^−4^) was gained for VTMoV that infects *Nicotiana velutina*, a native tobacco wild species of Australia [221].

It was suggested earlier that recombinational shuffling of the protease, RdRp and CP encoding genes during RNA replication—considered as “a modular evolution”—has been a key mechanism in the evolution of the “supergroup” involving the members of genus *Sobemovirus* and the families of *Luteoviridae* and *Tombusviridae* [222]. While sobemoviral polyprotein (Pro-VPg-RdRp) shows sequence similarity to that of enamo- and poleroviruses from the family *Luteoviridae*, sobemoviral CP has been related to CPs of necroviruses from the family *Tombusviridae* [124,144]. The viruses of the genera *Polerovirus* and *Enamovirus* are classified into the family *Luteoviridae* according to their homology with the representatives of the genus *Luteovirus* at the CP level, whereas their RdRp-s are clearly distant from each other [220,223]. Instead of Pro-VPg, luteovirus genome encodes a protein P1 possessing helicase-like motifs [223]. The P1-RdRp-s of luteoviruses are most similar to those of dianthoviruses but also to those of umbraviruses [74,224], both classified now as members of the family *Tombusviridae* [3]. The supergroup obviously involves also *Mushroom bacilliform virus* (MBV), a single member of the family *Barnaviridae* that has a genome organization similar to sobemoviruses—except that its ORF1 is largely overlapping with ORF2 in alternate frame and there is no data on ORFx. MBV polyprotein (Pro-VPg-RdRp) sequence is related to those of sobemo-, polero-, and enamoviruses, but its CP is distantly related to that of carmoviruses [75,225]. Likewise, a naturally occurring polero-sobemovirus hybrid *Poinsettia latent virus* (PnLV), now classified as a sole member of the genus *Polemovirus*, belongs to this supergroup, as its genome 5′ half is phylogenetically related with that of poleroviruses whereas its CP sequence resembles that of sobemoviruses [226]. The most likely model suggests that the recombinations arose by strand switching near the sgRNA start sites during RNA replication in cells co-infected with two parental viruses [74].

Remarkably, recombination seems to be especially intrinsic for the members of the family *Luteoviridae*, where recombinant species are abundant [227]. The phylogenetic analysis data on the family *Tombusviridae* suggest that there exist interspecies recombinants between necro- and carmovirus species [228,229] as well as between tombus- and carmovirus species [230]. The phylogenetic studies conclude that sobemovirus species have evolved in the absence of interspecies recombination events [217,231]. Also, no recombination event was detected between CfMV and RGMoV under little or no selection pressure in experimentally co-infected plants [232]. Few intraspecies recombination events have been identified for RYMV after sequencing a large pool of RYMV isolates collected throughout Africa [233,234]. The recombinants were found from eastern Tanzania and from Pemba Island in Zanzibar. The RYMV recombinants from Pemba were closely related to the other recombinants occurring in mainland eastern Tanzania [234]. Eastern Tanzania is considered as the putative center of RYMV origin, exposing the highest diversity and a fully mixed spatial distribution of the RYMV strains [12]. Whereas the recombinations between RYMV strains were observed to take place both in 3′ UTR as well as within ORF2a and ORF2b, the interspecies recombination breakpoints in the representatives of the genera *Luteovirus* and *Polerovirus* were identified to cluster at gene boundaries [220].

## 14. A Proposal for the Creation of the Family *Sobemoviridae*

What has been considered characteristic for sobemoviruses since the initial recognition of the genus is their transmission by beetles. Therefore, it has been proposed that the assignment of the genus *Sobemovirus* to any viral family cannot be decided before the mechanisms of this rare type of transmission are at least partially clarified. In fact, not all sobemoviruses are transmitted by beetles. As previously explained, some sobemoviruses are for example transmitted by leafhoppers, grasshoppers, mirids, or aphids. Remarkably, when full-length sequences of putatively beetle-transmitted sobemoviruses are compared to those possibly transmitted by other insects, no identifiable difference can be noticed. Moreover, it has been shown that at least RYMV and SCMoV are transmitted by different mammals and also by routine farming operations. This means that the virus is mechanically transmitted by whatever means that causes wounding of host plants. Also, several sobemoviruses can be transmitted through soil.

A similar situation can be seen within the phylogenetically related family *Tombusviridae*, whose members are readily transmitted by mechanical inoculation. These viruses are often found in the surface of waters and soils from where they can be acquired without vectors’ assistance. Transmission by beetles or by the chytrid fungus in the genus *Olpidium* has also been reported for members of several genera. One isolate of *Maize chlorotic mottle virus* (MCMV), a machlomovirus, is even transmitted by thrips [235].

Differently, the members of *Luteoviridae* are exclusively transmitted by specific aphid vectors. Only enamovirus (*Pea enation mosaic virus-1*, PEMV-1) is transmitted mechanically and this property is dependent on its multiplication in cells co-infected with PEMV-2 (genus *Umbravirus*). Interestingly, aphid transmissibility can be lost after several mechanical passages [223].

The single member of the genus *Polemovirus* (PnLV) is distributed by grafting and vegetative propagation of the host plant, but its natural means of transmission is unknown. It has been suggested that transmission from soil may also occur [236]. Transmission of the single representative (MBV), of the *Barnaviridae* occurs horizontally via mycelium and possibly by basidiospores [237].

The mode of transmission is determined by the properties of viral CPs, e.g., the read-through region of CPs of the members of *Luteoviridae* contains the aphid-transmission factor. In the case of *Luteoviridae*, the transmission mode has been used as the main criterion for grouping the viruses with different types of RdRp-s together into the same family [223].

The classification into different genera within *Tombusviridae* family is determined by the subtypes of CPs, MPs, and RdRp-s. Due to the absence of a CP-encoding gene, the genus *Umbravirus* was not assigned to any family until recently. Now, it has been grouped into the family *Tombusviridae* according to the phylogenetic relationships between the RdRp-s of umbraviruses and the members of *Tombusviridae*. The RdRp-s are highly conserved among members of the family *Tombusviridae*, showing the greatest level of conservation within the read-through (or frameshift) portion. RdRp-s of the members of *Tombusviridae* can be divided into three major subgroups: one associated with the tombus-aureusvirus lineage, one associated with the carmo-machlomo-panicovirus lineage and a third one associated with the diantho-umbravirus lineage, showing similarities with the RdRp-s of luteoviruses [3]. These RdRp-s are very distant from the RdRp-s of sobemo-polerovirus lineage in evolutionary terms—the RdRp-s of the members of *Tombusviridae* and *Luteovirus* belong to flavivirus-like RdRp superfamily, whereas the RdRp-s of the genera *Sobemovirus*, *Polemovirus*, *Polerovirus*, *Enamovirus,* and *Barnavirus* belong to picornavirus-like RdRp superfamily [238].

**Figure 5:**
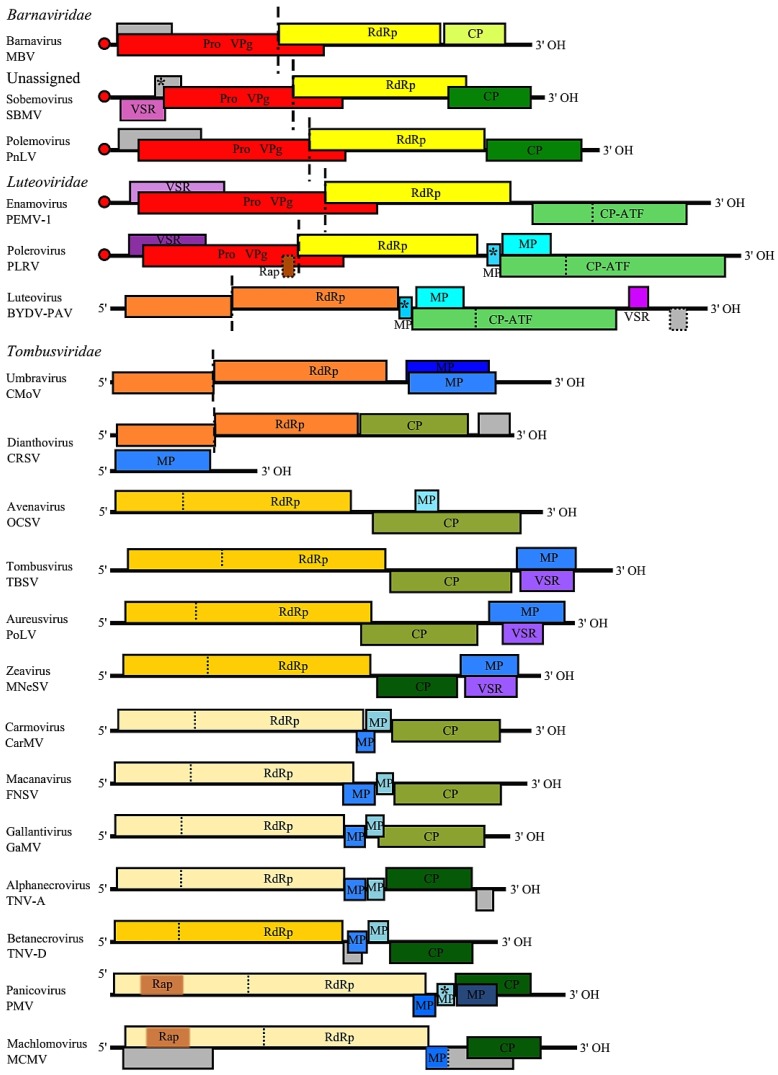
Genome annotations of the type species of phylogenetically related families and genera. NCBI species RefSeqs were used for making annotations. Polero- and luteovirus annotations were corrected according to [239]. Panicovirus annotation was corrected according to [240]. All genome annotations are shown to scale for size comparisons. RdRp genes are shown in the upper frame (frame 0); middle frame represents +1 frame and lower frame represents +2 frame. Non-canonical AUG start codon is marked with asterisk (*). –1 ribosomal frameshifting is marked by long vertical dashed line. Read-through codon is shown by short vertical dashed line. Boxes with dashed borders: unique for the type species. Different protein functions are displayed with different colors and the homology is indicated by different tones. Color code: bright yellow—picornavirus-like RdRp; soft yellows and orange—flavivirus-like RdRp (three subgroups within *Tombusviridae* family are shown); red—serine protease; green—CP (CPs of *Tombusviridae* are presented in two different tones to distinguish CPs with protruding domains (shown in olive green) and without it (shown in dark green)); blue—movement protein (MP); lilac or purple—viral RNAi suppressor (VSR); brown—replication-associated protein (Rap); grey—no function found. VPg is depicted as a red dot at the 5′ end of the genomic RNA. Virus name abbreviations: MBV—*Mushroom bacilliform virus* (NC_001633); SBMV—*Southern bean mosaic virus* (NC_004060); PnLV—*Poinsettia latent virus* (NC_011543); PEMV-1—*Pea enation mosaic virus-1* (NC_003629); PLRV—*Potato leafroll virus* (NC_001747); BYDV-PAV—*Barley yellow* *dwarf virus-PAV* (NC_004750); CMoV—*Carrot mottle virus* (NC_011515); CRSV—*Carnation ringspot* *virus* (NC_003530; NC_003531); OCSV—*Oat chlorotic stunt virus* (NC_003633); TBSV—*Tomato bushy stunt virus* (NC_001554); PoLV—*Pothos latent virus* (NC_000939); MNeSV—*Maize necrotic streak virus* (NC_007729); CarMV—*Carnation mottle virus* (NC_001265); FNSV*—Furcraea necrotic streak virus* (NC_020469); GaMV—*Galinsoga mosaic virus* (NC_001818); TNV-A—*Tobacco necrosis virus-A* (NC_001777); TNV-D—*Tobacco necrosis virus-D* (NC_003487); PMV—*Panicum mosaic virus* (NC_002598); MCMV—*Maize chlorotic mottle virus* (NC_003627).

Hence, there are two big families related to each other—*Luteoviridae* and *Tombusviridae*—but grouped using different main criteria (CP or RdRp homology) as argument for a membership (Figure 5). The genus *Sobemovirus* has phylogenetic relationships with both of them, but it cannot be assigned into one or another family, as it does not share homology with *Luteoviridae* at the CP level or with *Tombusviridae* at the RdRp level. Moreover, genomic sequences of individual sobemovirus species cluster very tightly together. Thus, it is impossible to assign the genus *Sobemovirus* to any existing viral family.

In addition to the RdRp phylogeny, the monophyly of the picornavirus-like superfamilies is supported by the conservation of both chymotrypsin-like protease and RdRp [238]. Using the existence of these two genes as the main criterion for taxonomical classification, we propose that the International Committee on Taxonomy of Viruses (ICTV) recognizes the novel plant virus family *Sobemoviridae* and we consider that the yet unassigned genera *Sobemovirus* and *Polemovirus* should be placed into this new family. The rationale for including *Polemovirus* is that although this genus is the product of a recombination between sobemoviruses and poleroviruses, it is still most closely related to *Sobemovirus* than to any other genus in the virosphere (Figure 5). A potential re-assignment of other viruses having the sobemo-like Pro-VPg-RdRp-s would need further discussion.

## 15. Perspectives

Sobemoviruses with their small icosahedral particles and single-stranded plus-oriented single-component compact RNA genomes are interesting but not unique. There are several viruses that have these same features, like the members of the families *Luteoviridae* and *Tombusviridae* (except the bipartite dianthoviruses). Sobemoviruses, once being an attractive model for structural biologists, remained molecularly poorly characterized by the end of the last century. Since then, as described in this review, we have gained a lot of new knowledge on the sequence of different sobemoviruses, on processing and functions of individual sobemoviral proteins, on resistance mechanisms against these viruses, on the evolution of sobemoviruses, *etc.* Still, much more is to be done. For instance: what are the molecular mechanisms for the dualistic function of P1 protein; what is the function of Px; why does the N-terminus of the polyprotein contain the putative membrane anchor; what are the cleavage sites of the polyprotein processing for different sobemoviruses; which processes is VPg actually needed for; what are the *in vivo* functions of P10 and P8 and are they expressed by each sobemovirus; which is the complex needed by sobemoviral RdRp to initiate the plus-strand and minus-strand synthesis; is this complex the same for sgRNA synthesis; how is it determined whether the virus enters the phloem or xylem? In addition, there is a lot to be done to elucidate whether the gigantic work made by D. Fargette’s group and their colleagues in Africa on RYMV evolution and resistance mechanisms is valid also for other sobemoviruses or not.
